# Electrical stimulation of the lateral cerebellar nucleus promotes neurogenesis in rats after motor cortical ischemia

**DOI:** 10.1038/s41598-020-73332-5

**Published:** 2020-10-06

**Authors:** Zheng Wu, Fangling Sun, Zijie Li, Min Liu, Xin Tian, Deyu Guo, Penghu Wei, Yongzhi Shan, Tingting Liu, Min Guo, Zixin Zhu, Wenrong Zheng, Yufeng Wang, Guoguang Zhao, Wen Wang

**Affiliations:** 1 Department of Experimental Animal Laboratory, Xuan-Wu Hospital of Capital Medical University, 45 Changchun Street, Beijing, 100053 China; 2 Department of Neurosurgery, Xuan-Wu Hospital of Capital Medical University, 45 Changchun Street, Beijing, 100053 China; 3 Department of Experimental Animal Laboratory, Beijing Geriatric Medical Research Center, 45 Changchun Street, Beijing, 100053 China

**Keywords:** Neurogenesis, Regeneration and repair in the nervous system, Stroke, Neurosurgery

## Abstract

Deep brain stimulation (DBS) has been tentatively explored to promote motor recovery after stroke. Stroke could transiently activate endogenous self-repair processes, including neurogenesis in the subventricular zone (SVZ). In this regard, it is of considerable clinical interest to study whether DBS of the lateral cerebellar nucleus (LCN) could promote neurogenesis in the SVZ for functional recovery after stroke. In the present study, rats were trained on the pasta matrix reaching task and the ladder rung walking task before surgery. And then an electrode was implanted in the LCN following cortical ischemia induced by endothelin-1 injection. After 1 week of recovery, LCN DBS coupled with motor training for two weeks promoted motor function recovery, and reduced the infarct volumes post-ischemia. LCN DBS augmented poststroke neurogenetic responses, characterized by proliferation of neural progenitor cells (NPCs) and neuroblasts in the SVZ and subsequent differentiation into neurons in the ischemic penumbra at 21 days poststroke. DBS with the same stimulus parameters at 1 month after ischemia could also increase nascent neuroblasts in the SVZ and newly matured neurons in the perilesional cortex at 42 days poststroke. These results suggest that LCN DBS promotes endogenous neurogenesis for neurorestoration after cortical ischemia.

## Introduction

Stroke is a global cause of death and neurological disability, accounting for almost 5% of disability-adjusted life-years and 10% of all deaths worldwide^1^. Although many patients survive the onset of stroke, most of them suffer from impaired quality of life due to residual functional deficits and disturbances. Remarkably, motor impairment of the upper limb occurs in 73–88% of first-time stroke survivors and in 55–75% of chronic stroke patients^2^. Stroke rehabilitation is a complex process, and the main hurdle for its improvement is the fragmentary knowledge about the physiological mechanisms impacting on functional brain reorganization and stroke recovery^3^. Novel neuromodulation modalities are urgently needed to augment current rehabilitative approaches.

Brain stimulation-based intervention, especially deep brain stimulation (DBS), may offer the opportunity for functional recovery to stroke victims, even those with severe motor impairment^4^. Most DBS treatments for poststroke disorders have focused on neuropathic pain, but only a handful of case reports exist on in the utility of DBS for treating motor deficits^5,6^. Nevertheless, these limited data do at least suggest that DBS may improve motor function following stroke. Recent studies have demonstrated that stimulation of the lateral cerebellar nucleus (LCN) using electrical or optogenetics approaches can promote motor function recovery after stroke^7,8^. In light of the capability for DBS to influence large cortical networks via modulation of intrinsic pathways, the mechanism of DBS in poststroke rehabilitation is still unclear.

Endogenous poststroke neurogenesis in the subventricular zone (SVZ) of the adult brain has been confirmed in neurological recovery, although it appears to be transient and insufficient to compensate for neuronal loss^9–11^. It is of significance that silenced neural stem cells (NSCs) can be activated using cellular and molecular approaches to proliferate, differentiate and migrate to an infarct area, which is technique we have been developing for years^12,13^. Hence, in the present study, we examine whether LCN DBS regulates neurogenesis in the ipsilesional and contralesional SVZ and whether it influences subsequent differentiation into neurons in perilesional cortex.

## Results

### Effects of LCN DBS on motor function recovery

**Figure 1:**
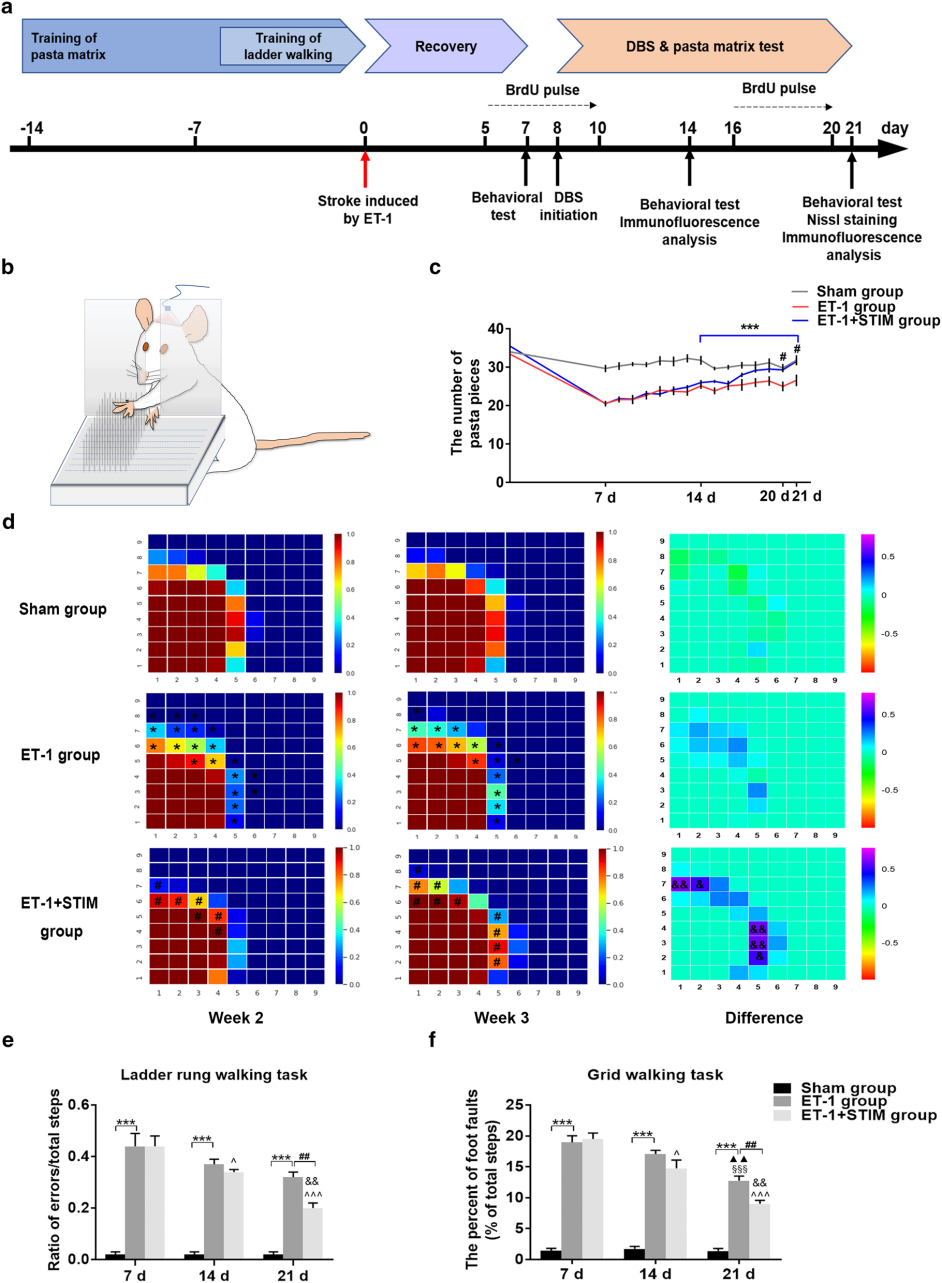
LCN DBS promoted motor function recovery. (**a**) Experimental schedule for DBS followed by a 1-week recovery. Stroke induction and electrode implantation were performed at day 0. Before that, during pre-surgery weeks, rats received training. DBS was started at 8 days post-surgery and continued for 2 weeks as described. (**b**) Pasta matrix reaching task for assessments. (**c**) Day-by-day analysis of pasta matrix reaching task performance (n = 6 for sham group and ET-1 + STIM group, n = 7 for ET-1 group, ^#^*P* < 0.05 compared to the ET-1 group. Data were analyzed by repeated measures ANOVA.). (**d**) Graphic representation of pasta retrieval at each location averaged over 7 days of testing for each group. The right side of pasta matrix was viewed from above. The bottom left corner of each figure represents the location of the slot where the rat’s paw can reach out towards the anterior and lateral matrix. Color coding bar indicates the frequency of pasta acquisition at each site (n = 6 for sham group and ET-1 + STIM group, n = 7 for ET-1 group, ^*^P < 0.05 compared to sham group, ^#^*P* < 0.05 compared to ET-1 group. Data were analyzed with the Mann–Whitney U-test. ^&^*P* < 0.05, ^&&^*P* < 0.01 between week 2 and week 3. Data were analyzed with the Student's t-test.). (**e**) Performance in ladder rung walking task at 7 days, 14 days and 21 days poststroke (n = 6 for sham group and ET-1 + STIM group, n = 7 for ET-1 group, ^***^P < 0.001 compared to sham group, ^##^*P* < 0.01 compared to ET-1 group, ^^^*P* < 0.05, ^^^^^*P* < 0.001 compared to ET-1 + STIM group at 7 days, ^&&^*P* < 0.01 compared to ET-1 + STIM group at 14 days.). (**f**) Performance in grid walking task at 7 days, 14 days and 21 days poststroke (n = 6 for sham group and ET-1 + STIM group, n = 7 for ET-1 group, ^***^P < 0.001 compared to sham group, ^##^*P* < 0.01 compared to the ET-1 group, ^§§§^*P* < 0.001 compared to ET-1 group at 7 days, ^▲▲^*P* < 0.01 compared to ET-1 group at 14 days, ^^^*P* < 0.05, ^^^^^*P* < 0.001 compared to ET-1 + STIM group at 7 days, ^&&^*P* < 0.01 compared to ET-1 + STIM group at 14 days .). Data in (**e**) and (**f**) were compared by one-way ANOVA with Scheffe’s multiple comparison test. Data are expressed as mean ± S.E.M.

We assessed the motor function of animals’ affected forelimbs at different time points by using the pasta matrix reaching task, the ladder rung walking task and the grid walking task (Fig. 1a). Figure 1b showed how the animals carried out the pasta matrix reaching task. The results from the pasta matrix reaching task showed that ET-1-induced stroke caused a significant decline in reaching capability of rat’s forelimbs compared to the capability of the sham group. However, after 13 days of stimulation, animals made more successful reach attempts compared to the ET-1 group (*P* = 0.0331, Fig. 1c). Furthermore, the average number of pasta pieces in the ET-1 + STIM group was 19% higher than that of the ET-1 group at 21 days poststroke (*P* = 0.0480, Fig. 1c). Analysis of the spatial pattern from pasta matrix revealed that animals receiving LCN DBS obtained more pasta pieces that were located further anteriorly and laterally than did the ET-1 group (Fig. 1d). Additionally, after 2 weeks of stimulation, the performance of the ET-1 + STIM group in the ladder rung walking task was improved more clearly than that of the ET-1 group (*P* = 0.0023, Fig. 1e), and the percent of foot faults in the grid walking task was significantly reduced at 21 days poststroke, compared to the ET-1 group (*P* = 0.0021, Fig. 1f). Furthermore, the performance of the ET-1 + STIM group in all the three behavioral tests at 21 days were significantly improved than that at 14 days poststroke (*P* = 0.0005, Fig. 1c; *P* = 0.0057, Fig. 1e; *P* = 0.0036, Fig. 1f), while statistical differences were only observed in the grid walking task of the ET-1 group between 14 and 21 days poststroke (*P* = 0.0069, Fig. 1f). The results described above suggest that the motor function of impaired forelimbs can be improved by LCN DBS coupled with motor rehabilitation after 1 week of recovery.

### Effects of LCN DBS on infarct volume

**Figure 2:**
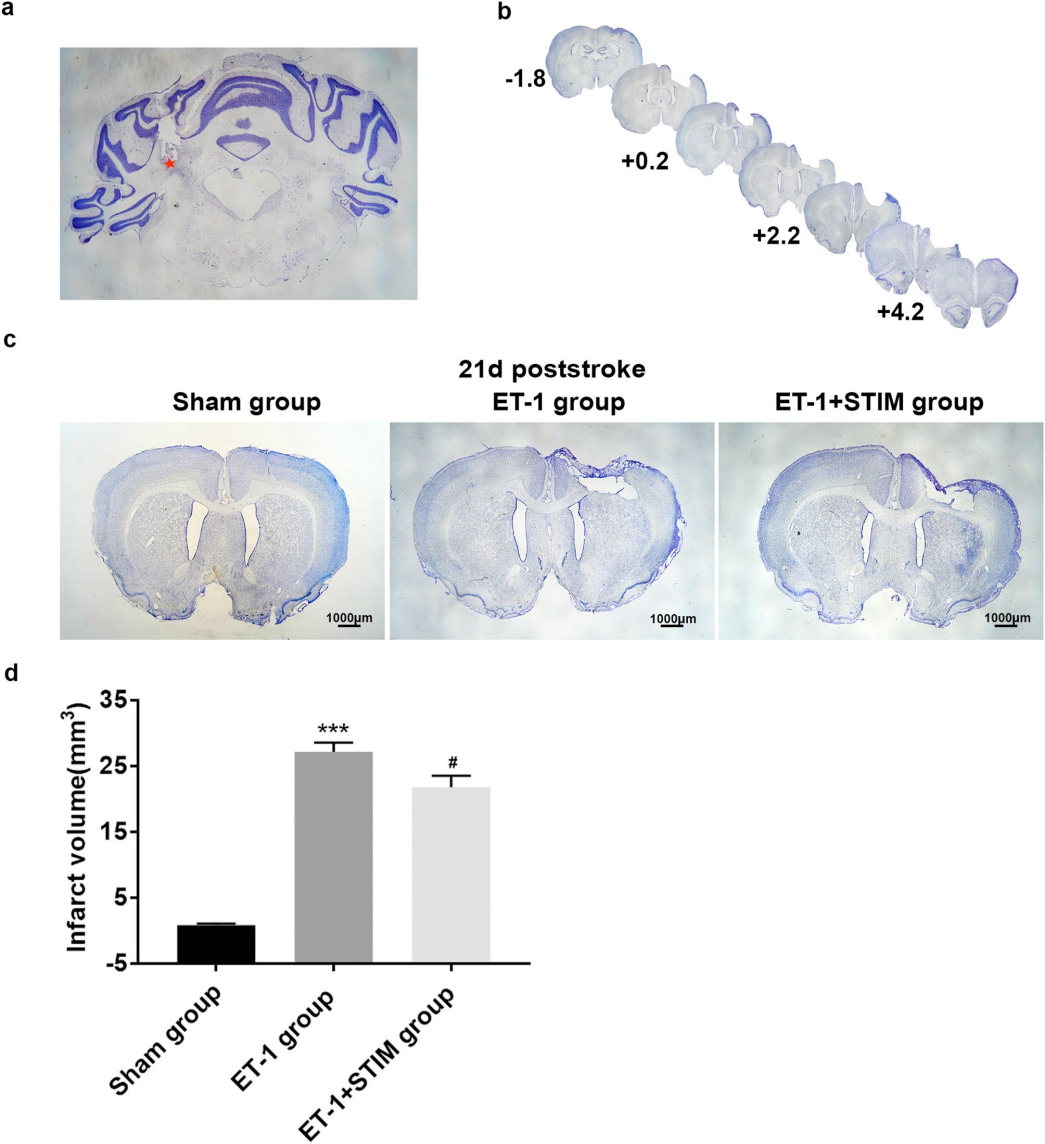
LCN DBS reduced infarct volume. (**a**) Star indicates the location of the implanted electrodes in the dorsal LCN. (**b**) Infarct volume calculated from 4.2 mm anterior to 1.8 mm posterior to bregma. (**c**) Representative images of Nissl staining of three groups at 21 days poststroke. (**d**) Quantitative analysis of infarct volume (n = 5 for each group, ^***^P < 0.001 compared to sham group, ^#^*P* < 0.05 compared to ET-1 group. One-way ANOVA followed by Scheffe’s multiple comparison test was used to analyze data). Data are expressed as mean ± S.E.M.

Nissl staining was used to detect the electrode location and infarct area as shown in Fig. 2a and b. After 2 weeks of stimulation, the infarct volume for the ET-1 + STIM group was 21.78 ± 1.73 mm^3^, which was 19.75% lower than that of the ET-1 group (27.14 ± 1.43 mm^3^) (*P* = 0.0413, Fig. 2c and d), suggesting that LCN DBS at 1 week after ischemia reduced infarct volume.

### Effects of LCN DBS on the proliferation of neural progenitor cells (NPCs) and neuroblasts in the SVZ at 14 days and 21 days poststroke

**Figure 3:**
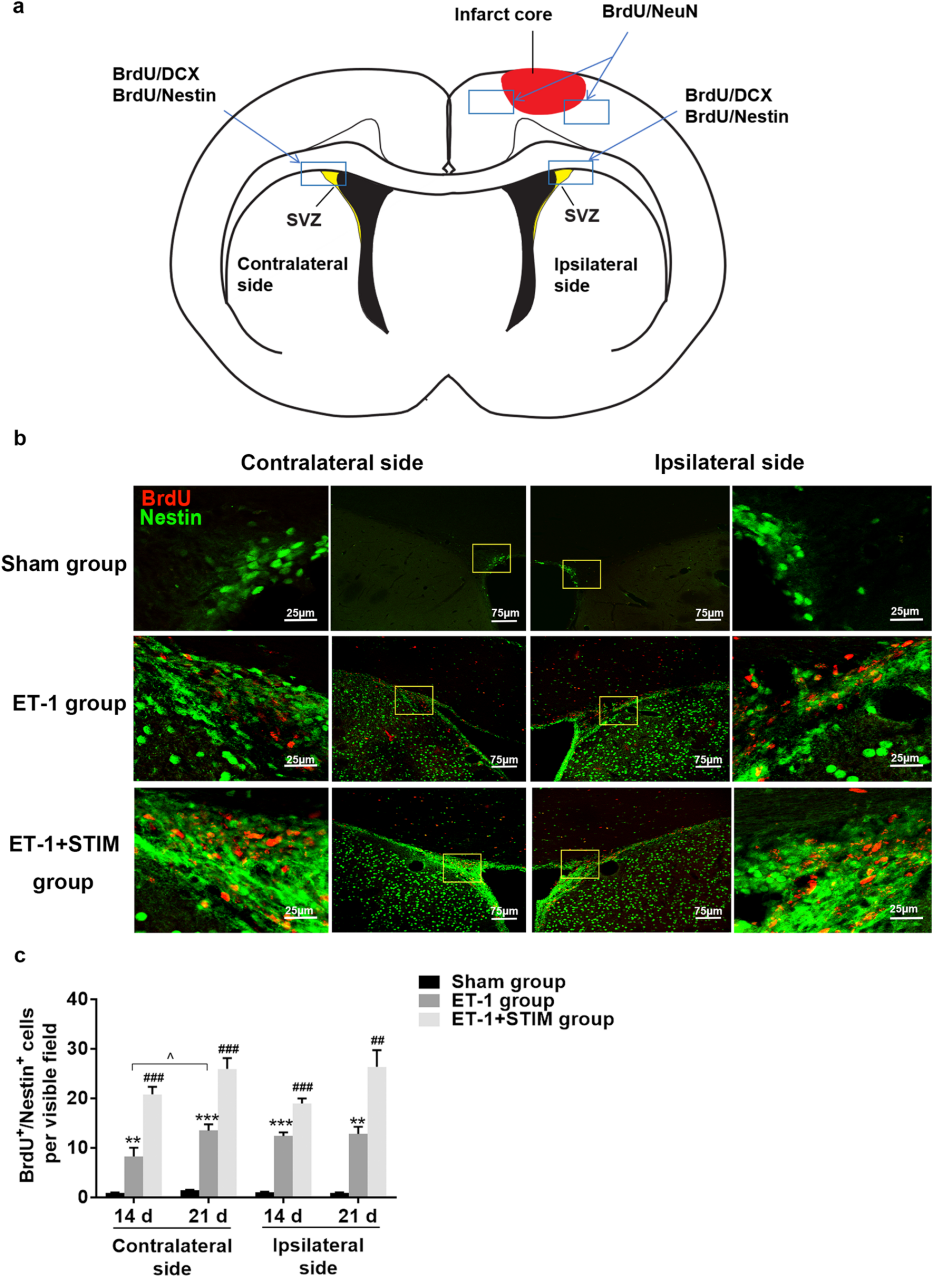
LCN DBS promoted the proliferation of NPCs in the SVZ. (**a**) The locations of areas of interest in the SVZ and in perilesional cortex for immunofluorescence analysis. (**b**) Representative images of BrdU^+^/Nestin^+^ cells in three groups. Yellow boxes magnified in each adjacent image. (**c**) Quantitative analysis of BrdU^+^/Nestin^+^ cells per visible field in the ipsilesional and contralesional SVZ at 14 days and 21 days poststroke (n = 5 for each group at 14 days, ^**^P < 0.01, ^***^P < 0.001 compared to sham group, ^###^*P* < 0.001 compared to ET-1 group. One-way ANOVA followed by Scheffe’s multiple comparison test was used to analyze data; n = 5 for each group at 21 days, ^**^P < 0.01, ^***^P < 0.001 compared to sham group; ^##^*P* < 0.01, ^###^*P* < 0.001 compared to ET-1 group. One-way ANOVA followed by Scheffe’s multiple comparison test was used to analyze data; ^^^*P* < 0.05 compared to ET-1 group at 14 days. The data of BrdU^+^/Nestin^+^ cells at 14 days and at 21 days were compared by Student's t-test.). Data are expressed as mean ± S.E.M.

**Figure 4:**
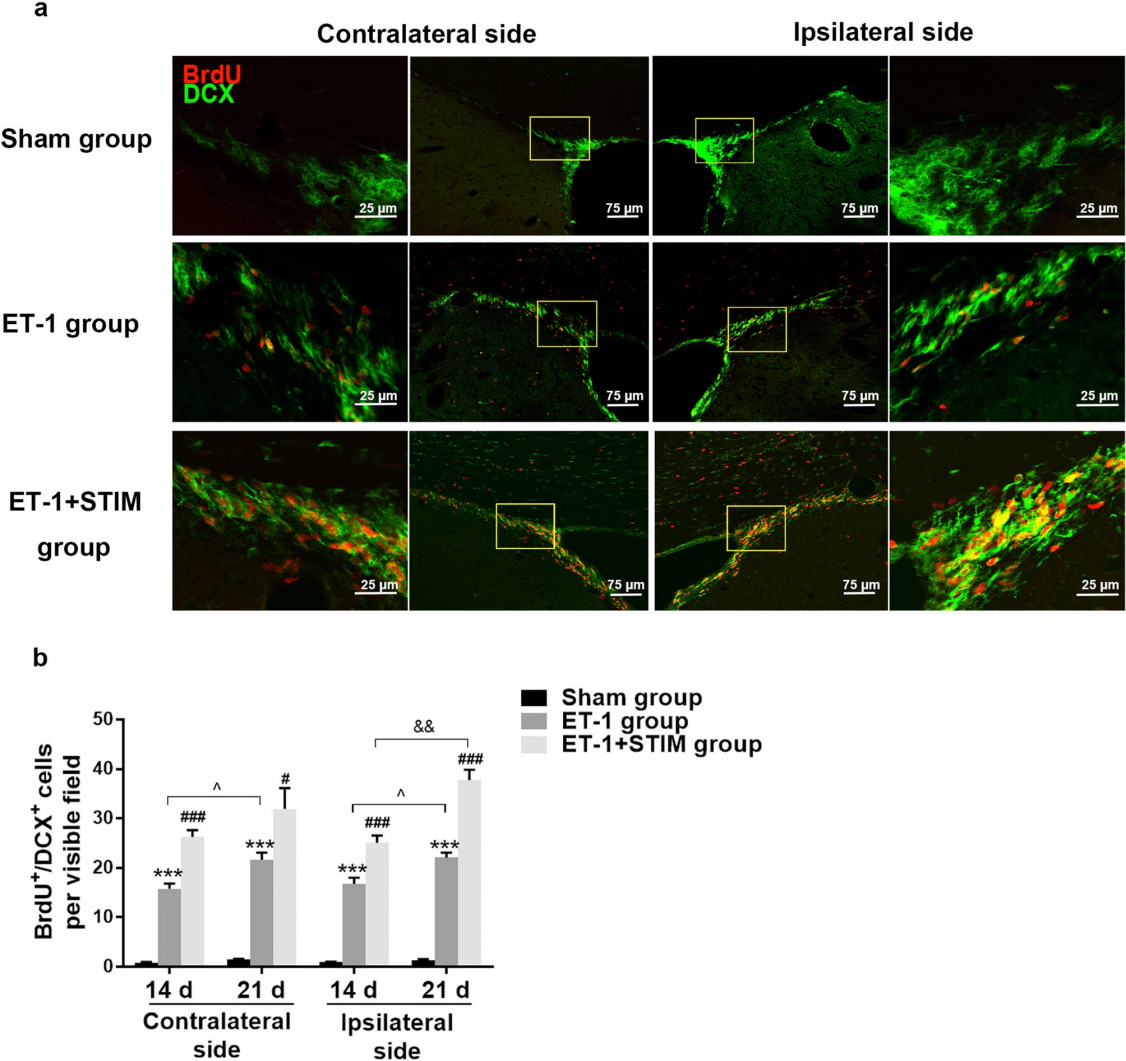
LCN DBS increased the number of new neuroblasts in the SVZ. (**a**) Representative immunofluorescent staining images of BrdU^+^/DCX^+^ cells in three groups. (**b**) Quantitative analysis of BrdU^+^/DCX^+^ cells at 14 days and at 21 days poststroke (n = 5 for each group at 14 days, ^***^P < 0.001 compared to sham group, ^###^*P* < 0.001 compared to ET-1 group. One-way ANOVA followed by Scheffe’s multiple comparison test was used to analyze data; n = 5 for each group at 21 days, ^***^P < 0.001 compared to sham group, ^#^*P* < 0.05, ^###^*P* < 0.001 compared to ET-1 group. One-way ANOVA followed by Scheffe’s multiple comparison test was used to analyze data; ^^^ *P* < 0.05 compared to ET-1 group at 14 days; ^&&^ *P* < 0.01 compared to ET-1 + STIM group at 14 days. The data of BrdU^+^/DCX^+^ cells at 14 days and at 21 days were compared by Student's t-test.). Data are expressed as mean ± S.E.M.

To investigate the effects of LCN DBS on neurogenesis poststroke, we observed BrdU^+^/Nestin^+^ and BrdU^+^/DCX^+^ cells in the areas of interest in the SVZ (Fig. 3a). As shown in Fig. 3b, BrdU^+^ cells colocalization with Nestin in the ipsilesional and contralesional SVZ was observed. We found that the double-positive cells of the ET-1 group increased significantly compared to that of the sham group at 14 days (ipsilesional SVZ: *P* = 0.0000, contralesional SVZ: *P* = 0.0086, Fig. 3c) and 21 days (ipsilesional SVZ: *P* = 0.0064, contralesional SVZ: *P* = 0.0003, Fig. 3c) poststroke. Furthermore, after 2 week of stimulation treatment, the numbers of BrdU^+^/Nestin^+^ cells on both sides of the SVZ were significantly elevated on day 21 poststroke (ipsilesional SVZ: *P* = 0.0027, contralesional SVZ: *P* = 0.0003, Fig. 3c). Numbers for the ET-1 + STIM group did not significantly differ from those for 1 week of stimulation. Co-expression of DCX and BrdU was also detected in the ipsilesional and contralesional SVZ (Fig. 4a). Consistent with the results for the BrdU^+^/Nestin^+^ cells, at 14 days poststroke, there was a marked increase in BrdU^+^/ DCX^+^ cells by cortex ischemia compared to that of the sham group (ipsilesional SVZ: *P* = 0.0000, contralesional SVZ: *P* = 0.0000, Fig. 4b), and LCN DBS treatment more strongly enhanced co-expression than for non-treatment (ipsilesional SVZ: *P* = 0.0008, contralesional SVZ: *P* = 0.0000, Fig. 4b). The promoting effect of LCN DBS was sustained until 21 days poststroke (ipsilesional SVZ: *P* = 0.0000, contralesional SVZ: *P* = 0.0468, Fig. 4b), and was further enhanced compared to that of DBS for 1 week (ipsilesional SVZ: *P* = 0.0010, Fig. 4b). These results suggest that LCN DBS can enhance neurogenesis in the ipsilesional and contralesional SVZ, thereby facilitating the process of restoration after ischemia.

### Effects of LCN DBS on neuronal differentiation in perilesional cortex at 21 days poststroke

**Figure 5:**
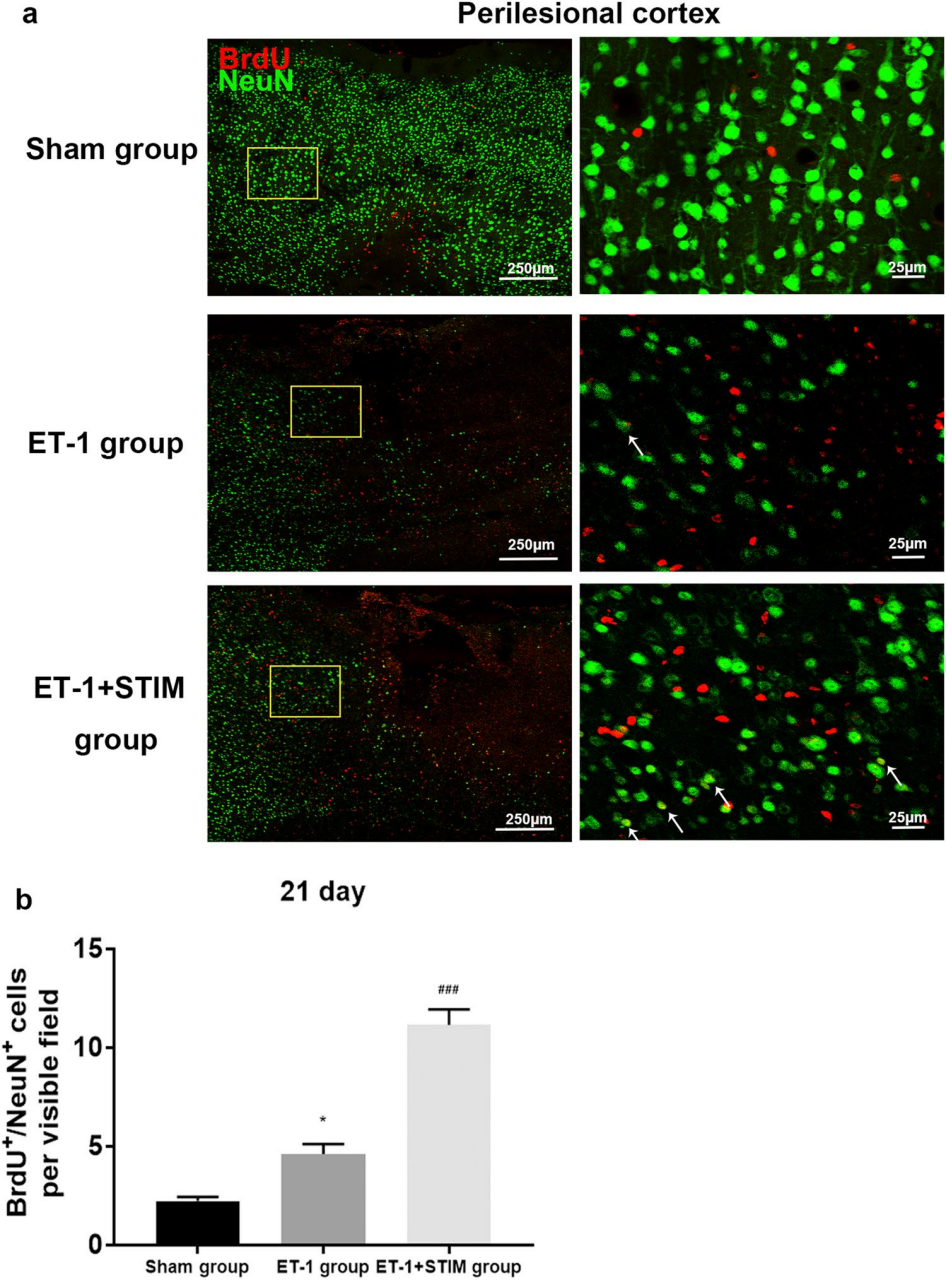
LCN DBS promoted neuronal differentiation in perilesional cortex. (**a**) Representative immunofluorescent staining images of BrdU^+^/NeuN^+^ cells in three groups. Arrowheads indicate co-expression of BrdU and NeuN. (**b**) Quantitative analysis of BrdU^+^/NeuN^+^ cells in perilesional cortex at 21 days poststroke (n = 5 for each group, ^*^P < 0.05 compared to sham group, ^###^*P* < 0.001 compared to ET-1 group. One-way ANOVA followed by Scheffe’s multiple comparison test was used to analyze data). Data are expressed as mean ± S.E.M.

After stroke, in perilesional cortex, some of neurons stained with NeuN displayed shrunken cytoplasm and pyknotic nuclei, while some retaining cellular integrity and intact nuclei were observed occasionally co-labeled with BrdU (Fig. 5a). After 2 weeks of stimulation, the number of BrdU^+^/NeuN^+^ cells increased to more than two-fold by 21 days (*P* = 0.0001, Fig. 5b), indicating that LCN DBS can enhance neuronal differentiation in perilesional cortex. Few BrdU^+^/NeuN^+^ cells were detected around infarct zones at 14 days with 1 week of stimulation (data not shown).

### Effects of LCN DBS on neurogenesis at 42 days poststroke

**Figure 6:**
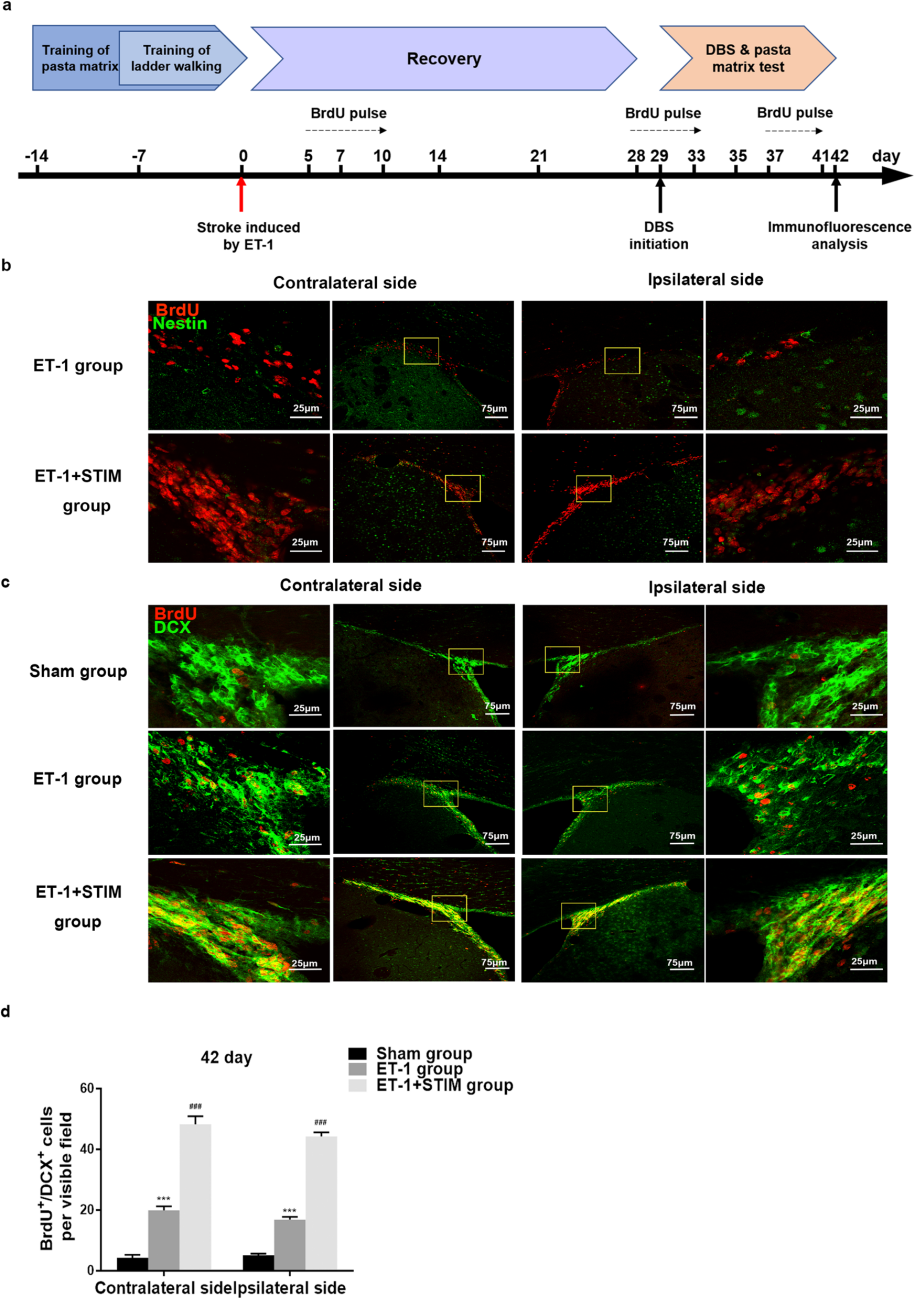
LCN DBS increased the number of new neuroblasts at 42 days poststroke. (**a**) An overview of experimental design for DBS followed by a 4-week recovery. DBS was started at 29 days post-surgery and continued for 2 weeks as described. (**b**) Representative immunofluorescent staining images of BrdU^+^/Nestin^+^ cells in ET-1 group and ET-1 + STIM group. (**c**) Representative images of BrdU^+^/DCX^+^ cells in three group. (**d**) Quantitative analysis of BrdU^+^/DCX^+^ cells in the SVZ at 42 days poststroke (n = 5 for each group, ^***^P < 0.001 compared to sham group, ^###^*P* < 0.001 compared to ET-1 group. One-way ANOVA followed by Scheffe’s multiple comparison test was used to analyze data). Data are expressed as mean ± S.E.M.

**Figure 7:**
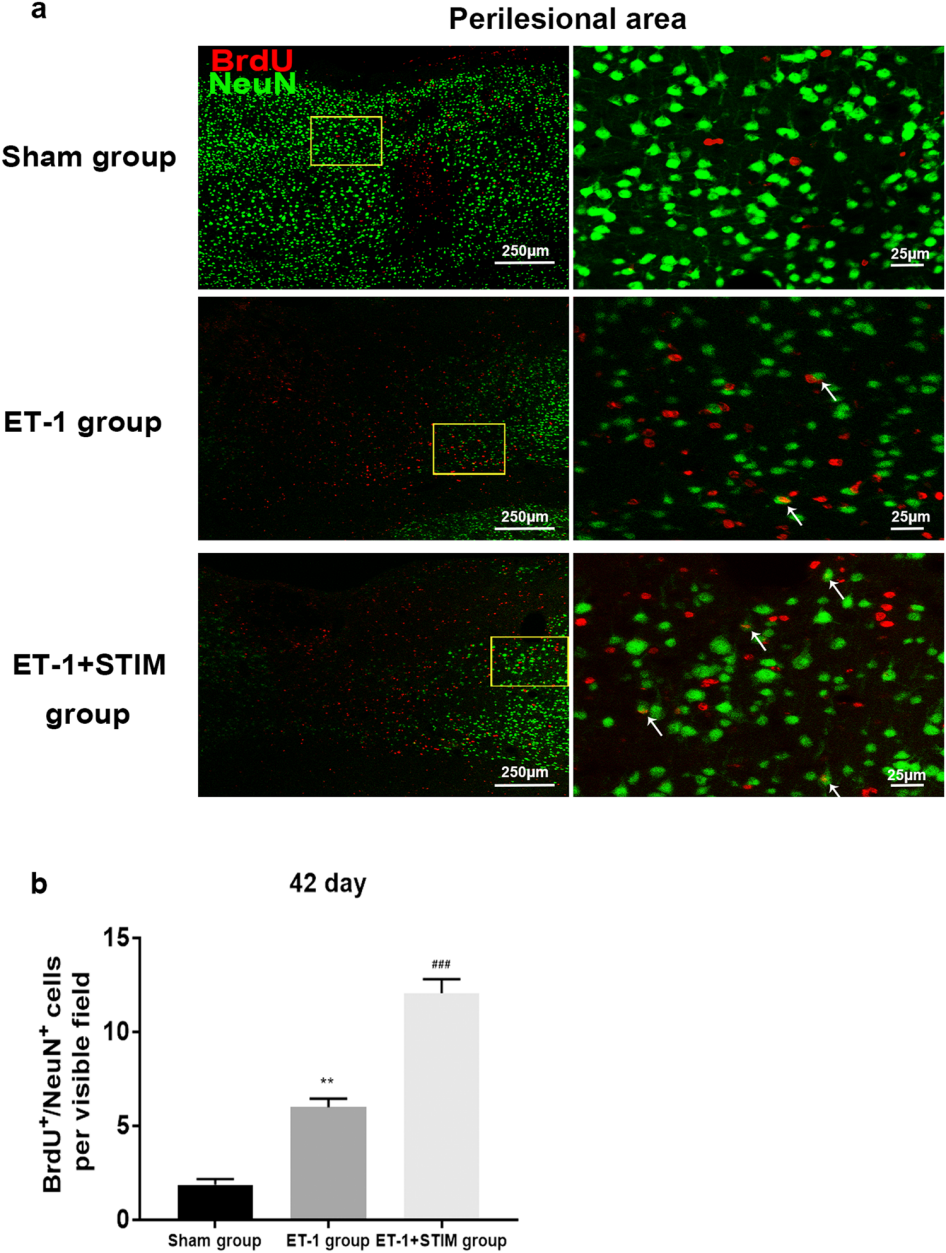
LCN DBS promoted neuronal differentiation in perilesional cortex at 42 days poststroke. (**a**) Representative immunofluorescent staining images of BrdU^+^/NeuN^+^ cells in three groups. (**b**) Quantitative analysis of BrdU^+^/NeuN^+^ cells in perilesional cortex at 42 days poststroke (n = 5 for each group, ^**^P < 0.01 compared to sham group, ^###^*P* < 0.001 compared to ET-1 group. One-way ANOVA followed by Scheffe’s multiple comparison test was used to analyze data). Data are expressed as mean ± S.E.M.

We further examined BrdU^+^/Nestin^+^, BrdU^+^/DCX^+^ and BrdU^+^/NeuN^+^ cells at 42 days poststroke after 4 weeks of recovery and 2 weeks of stimulation (Fig. 6a). Figure 6b indicates there were few BrdU^+^/Nestin^+^ cells in the ipsilesional and contralesional SVZ of rats in the ET-1 group at 42 days poststroke, yet 2 weeks of stimulation did not significantly increase numbers of double positive cells. However, the numbers of BrdU^+^/DCX^+^ cells in the ipsilesional and contralesional SVZ of the ET-1 + STIM group were respectively increased to 44.28 ± 1.36 and 48.25 ± 2.67 versus 16.88 ± 0.88 and 19.95 ± 1.30 in the ET-1 group at 42 days poststroke (ipsilesional SVZ: *P* = 0.0000, contralesional SVZ: *P* = 0.0000, Fig. 6c and d), indicating the promoting effect of delayed 2 weeks of stimulation on new neuroblasts. Moreover, after 4 weeks of recovery, LCN DBS also increased BrdU^+^/NeuN^+^ cells in perilesional cortex (*P* = 0.0000, Fig. 7a and b), benefiting self-neurorestoration.

## Discussion

Upper limb function is one of the best predictors of long-term disability after stroke. Nevertheless, despite extensive therapy, recovery is frequently incomplete. Improved effective strategies to promote functional recovery are urgently needed. Although invasive cortical stimulation has once been regarded as a potential rehabilitation therapy for stroke, it is still controversial since a Phase III trial has shown no advantage on reducing disability^14,15^. As another form of invasive brain electrode stimulation, DBS has provided great benefits for stroke rehabilitation, especially on improving motor recovery poststroke by locally and remotely targeting intrinsic neural circuits^4^.

Neuroimaging and electrophysiological studies have demonstrated the particular role of the dentate-thalamo-cortical (DTC) tract in the elaboration of complex movements. Through this pathway, the lateral cerebellum sends major excitatory output to contralesional motor areas, such as the primary motor and ventral premotor cortex as well as nonmotor areas such as the dorsolateral prefrontal cortex^16,17^. Selectively stimulating neurons of the contralesional LCN using an optogenetics approach was recently reported to promote robust and persistent recovery after stroke^8^. Hence, LCN may be a promising candidate for a DBS target for neuromodulation poststroke. Recent studies demonstrated that chronic electrical stimulation of the LCN can enhance motor recovery, promote cortical plasticity, and suppress neuroinflammation^18,19^, and a single-center study has been evaluating the safety and patient outcomes of LCN DBS for the management upper extremity hemiparesis due to ischemic stroke^20^. In the present study, we used three behavioral methods to demonstrate the improvement in affected limb function. The results confirmed that 30 Hz LCN DBS can improve motor function of rats’ forelimb poststroke. Furthermore, this enhanced motor recovery was associated with reduced infarct volume after DBS therapy. Thus, DBS has significant translational potential in brain repair after stroke, as a safe technique extensively used in clinics. However, there is currently a lack of evidence of how LCN DBS influences motor recovery. The present work aimed to evaluate the possible mechanisms related to the beneficial effects.

The SVZ and the dentate gyrus of the hippocampus have been verified to be two major niches where NSCs are generated in the adult brain^21^. Accumulating evidence have indicated that NPCs and neuroblasts in the SVZ migrated into the ischemic penumbra of the adjacent striatum and into ischemic cortex via the rostral migratory stream and lateral cortical stream^9,22,23^. Our group is interested in exploring effective approaches to persistently augment endogenous neurogenesis for functional recovery after stroke. Based on the hypothesis of neurovascular homeostasis rehabilitation (NHR) we proposed, exogenous approaches could promote neurogenesis, angiogenesis and blood–brain barrier (BBB) restoration to repair cerebral injury after stroke^12,13,24,25^. We assumed that DBS might be combined with physical rehabilitation or pharmacological interventions to effectively regulate neurogenesis for functional recovery in coming decades. The present study measured the BrdU^+^/Nestin^+^ cells and BrdU^+^/DCX^+^ cells related to ET-1-induced ischemia in the SVZ and found that the proliferation of NPCs can be enhanced by LCN DBS combined with pasta reaching training, initiated at week 2 poststroke. These effects appeared even when DBS therapy was applied for only 1 week. Interestingly, numbers of new NPCs and neuroblasts in the contralesional SVZ were also increased. This result is consistent with previous studies^26–28^, and further demonstrates the intrinsic connectivity between bilateral brain regions. Furthermore, it was supposed that the effects of DBS therapy on neurogenesis may rely on the mobilization stimulus of cortical ischemia. Activation of the DTC pathway could persistently enhance one or more certain mechanisms related to neurogenesis in the SVZ poststroke. In particular, a network of neurotrophic signals operating in an autocrine or paracrine manner may regulate neurogenesis in adult SVZ^29^. Unilateral LCN DBS acts in the context of an integrative tissue response, which may be transferred by CSF in ventricles or blood to the NPCs distributed along the lateral ventricular wall^11,30^. DBS combined with rehabilitation for 2 weeks also increased the number of nascent neurons labeled with BrdU and NeuN in the perilesional cortex. Similarly, the recent study by Chan et al. in 2018 showed DBS of the LCN after stroke had significant effects on both cortical and thalamic neurogenesis^18^. Unfortunately, they did not detect BrdU-labeled NPCs migrating from the SVZ or subgranular zone to the perilesional motor cortex 10 weeks after stroke induction. The difference in results might be due to the time points of the neurogenesis process. DBS initiated at week 6 poststroke in their study may miss the therapeutic window of neurogenesis in the SVZ, since most studies have revealed that nestin-positive NPCs increase during the month following ischemic stroke, and undergo degeneration during the post-acute stroke phase^28,31–33^. In addition, we further observed the effects on neurogenesis of DBS initiated at 5 weeks poststroke. The results showed few nascent NPCs in the SVZ 42 days poststroke, and DBS had no effects on the proliferation of BrdU^+/^Nestin^+^ cells. However, the positive effects on BrdU^+^/DCX^+^ cells and BrdU^+^/NeuN^+^ cells proved that LCN DBS could promote the differentiation of NPCs to neuroblasts and even to neurons. Another group in Japan observed that 1-week electrical stimulation of the striatum increased endogenous cell proliferation in the SVZ, followed by migration and differentiation of the newly formed cells into neurons^34^. This striatal DBS was initiated at the acute phase of brain injury and was comparatively extended to 1 month after ischemia, both within the time window of neurogenesis. One limitation remains, i.e., severe white matter lesion infiltration and the neuronal repair. In the present study, ET-1 injection induced marked white matter lesion, and the destruction of the subcortical white matter might contribute to limiting cortical migration. Thus, moderate motor cortical injury would be warranted to explore neurogenetic processes, by improving ET-1 injection methods.

**Figure 8:**
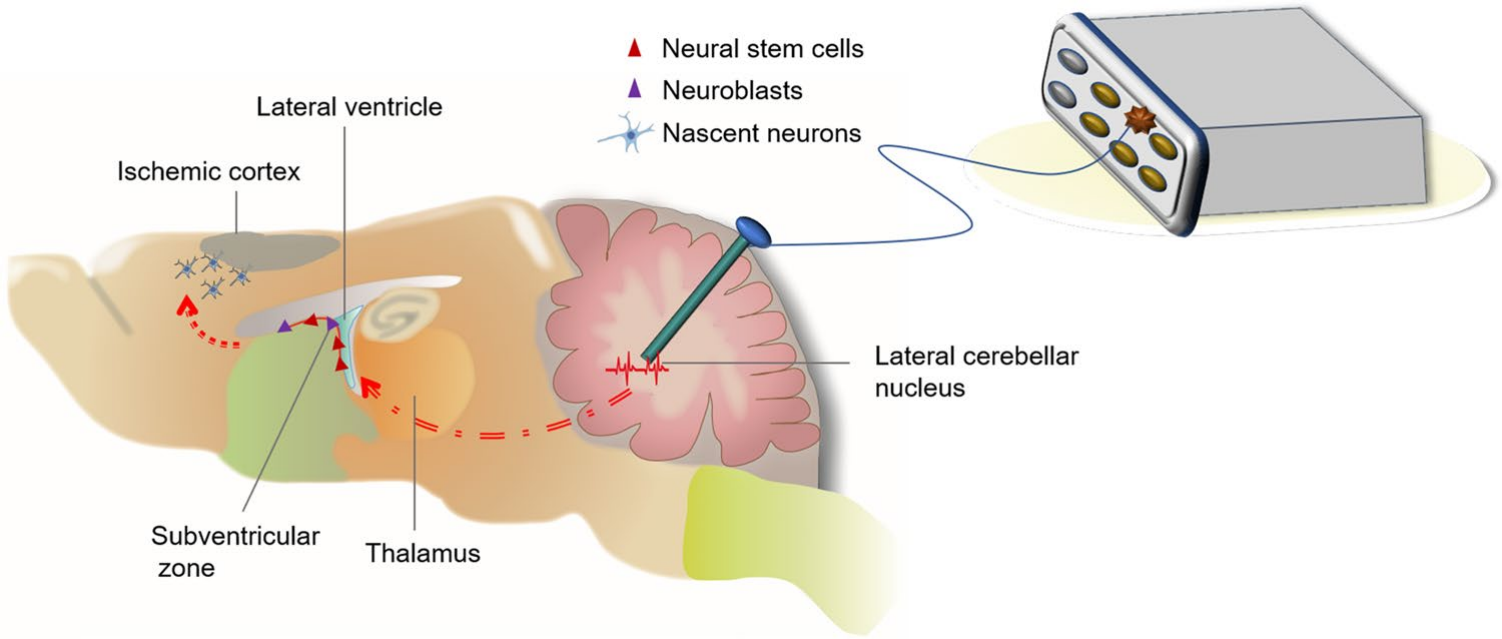
A possible strategy to stimulate endogenous brain repair process by LCN DBS for stroke recovery. Brain injury activates endogenous neurogenesis shortly after stroke. Neuromodulation approaches such as LCN DBS can promote a persistent increase in the number of NPCs from SVZ, and promote neuronal differentiation.

Although our observations of the proliferative NPCs are insufficient due to the limited time points and the lack of explicit mechanisms, these findings indicate a possible strategy to stimulate endogenous brain repair process for stroke recovery (Fig. 8). A previous study reported an increase in NPCs in the SVZ associated with subthalamic nuclei DBS in patients with Parkinson’s disease^35^. Preclinical studies have also demonstrated anti-epilepsy effects, antidepressant/anxiolytic responses and memory improvements by DBS correlated with neurogenesis^36–38^. Even so, adult neurogenesis is very species-specific, and the occurrence of neurogenesis in humans needs more critical thinking. Moreover, if neurogenesis occurs, it takes much longer than in rodents^22,39^. Together with these findings, we hope that endogenous stem cell proliferation and differentiation into intrinsic circuits can in the future be stimulated for the treatment of stroke and neurodegenerative diseases.

## Conclusion

The present study demonstrates that LCN DBS at 1 week or 1 month after brain ischemia promoted endogenous neurogenesis in the SVZ and then facilitated neuronal differentiation in perilesional cortex. These results provide evidence for potential neurorestoration effects of LCN DBS as a therapeutic tool in stroke rehabilitation.

## Methods

### Animals

Experiments were performed on 60 male Sprague–Dawley (SD) rats weighing 280–300 g, purchased from Beijing Vital River Experimental Animal Co. (Beijing, China). 45 animals were used for experiments of DBS followed by a 1-week recovery, and 15 animals were used for experiments of DBS followed by a 4-week recovery. The rats were housed under a 12/12 h dark/light cycle and specific-pathogen-free (SPF) conditions. All animal protocols for this study were conducted in accordance with the Administration Regulations on Laboratory Animals (Ministry of Science and Technology, 2017) and approved by the Animal Care and Use Committee of Xuanwu Hospital of Capital Medical University (Ethics Approval No.: 20180101).

### Endothelin-1-induced ischemia model

Male rats were anesthetized with isoflurane (5% induction in 70% nitrous oxide and 30% oxygen and maintenance with 2% isoflurane) and were then secured on a stereotaxic frame (David Kopf, California, USA). A midline incision was made to expose the calvaria, and three small burr holes were drilled at the coordinates given below: (1) (anterior–posterior, AP) = − 1.0 mm, (medial–lateral, ML) =  − 2.5 mm, (dorso-ventral, DV) =  − 2.3 mm; (2) AP =  + 1.0 mm, ML = − 2.5 mm, DV = − 2.3 mm; (3) AP =  + 3.0 mm, ML = − 2.5 mm, DV = − 2.3 mm^40^. ET-1 (Millipore, USA) was dissolved in sterile saline, and injected at 800 pmol/2 μL per hole. Sham-operated animals underwent the same surgical procedure, except that saline was injected instead of ET-1. After the surgery, each rat was placed on a 37 °C heating plate until fully awake from anesthesia and then returned to its cage.

### LCN deep brain stimulation

As shown in Figs. 1a and 6a, after 1 week/4 weeks of recovery poststroke, continuous LCN DBS was performed for 8 h per day combined with daily pasta reaching training with the affected paw. Rats were anesthetized with isoflurane (5% induction in 70% nitrous oxide and 30% oxygen and maintenance with 2% isoflurane), and were then secured on a stereotaxic frame (David Kopf, California, USA). A midline incision was made to expose the calvaria, and a small burr hole was drilled for implanting with homemade macro-electrodes in the LCN contralesional to the ET-1 injection sites at coordinates in relation to bregma: AP = − 11 mm, ML =  + 3.6 mm, DV = − 6.3 mm^40^. The electrodes were fabricated from PFA-insulated tungsten (diameter 175 μm, California Fine Wire, Grover Beach, CA) with an 1 mm exposure (below 1mΩ). Each electrode was fixed to the skull using dental acrylic and reinforced with three small screws set into the exposed skull. The implant locations in all animals were verified by using Nissl staining (Supplementary Figure 1), and the rats were included when the sections containing lesion site at bregma levels from − 10.6 to − 11.6 mm by comparing with corresponding slices in the rat brain atlas of Paxinos and Watson^41^. Besides, by increasing stimulation frequency, reproducible forelimb movements were induced to validate the accuracy of electrode location. Animals that did not exhibit visible motor response were excluded in the study. Isochronous stimulation at 30 Hz was controlled by a stimulator (AD Instruments, USA) at 80% of the motor threshold, with the pulse width maintained at 400 μs, ranging from 70 to 130 μA. The motor threshold for cerebellar stimulation was determined as the lowest current to induce visible motor response in ipsilateral forepaw, vibrissae or torso. Animals in the ET-1 group and the sham group were implanted with electrodes in a non-stimulation state.

### Pasta matrix reaching task

Animals were trained on the pasta matrix reaching task every day (three times per day: 8 a.m./12 a.m./7 p.m.) for 2 weeks before ET-1-induced stroke, according to previous studies^7,42^. During all behavioral testing periods, food was restricted to approximately 14.68 g/day. During training, pasta on both sides of the matrix was used to observe an animal’s dominant forepaw. The severity of impairment of animals’ affected forelimbs related to stroke was assessed by the pasta matrix reaching task before stimulation. Animals were divided into two groups: (1) no stimulation (ET-1 group) and (2) stimulation (ET-1 + STIM group). Assignments were made pseudorandomly to match severity of motor deficits for each group based on mean retrievals from pasta matrix poststroke, which facilitated the efficacy assessment of stimulation in a reasonable way, and avoided the influence of varying stroke severity on data outcomes. The rats were placed in the cage with a vertical slot in the front panel, through which the forelimb of rats reach the pasta pieces and break to remove it from half side of the pasta matrix (9*9 pieces) contralateral to the affected limb (Fig. 1b). During 2 weeks of stimulation, rats received pasta matrix task tests for 15 min per day for motor rehabilitation. The numbers and distribution of retrievals of pasta were recorded.

### Ladder rung walking task

Rats were videotaped as they walked along a horizontal ladder with irregular rung arrangement (length: 100 cm, width: 8 cm, 40 cm above the ground) to assess impairment in forelimb function after stroke^43^. After training for 1 week before surgery, each animal was allowed to cross the ladder in the same direction, which ended at the rat’s home cage for encouragement. The average of three measurements (7 days, 14 days and 21 days poststroke) was taken for each rat. Any slight paw slips, deep paw slips, and complete misses were scored as errors, and the results were expressed as ratios of errors to total steps.

### Grid walking task

For the grid walking task, rats were placed on an elevated grid surface (length: 100 cm, width: 10 cm, 50 cm above the ground) with grid openings of 9 cm^2^ at 7 days, 14 days and 21 days poststroke^44^. During locomotion on the grid, each animal completed three trials lasting 3 min, with intertrial intervals of 1 min. The grid walking task captured on video camera was replayed and analyzed for misplacement and slipping below by left forepaw on the grid.

### Infarct volume quantification

After rats were perfused with 0.9% saline and then 4% phosphate-buffered paraformaldehyde until limbs were stiffened, each brain was cryoprotected with 30% sucrose after being fixed in 4% phosphate-buffered paraformaldehyde. Brains were then sectioned in the coronal plane at 30 μm. Every 10th section containing infarcts at bregma levels from -1.80 to + 4.20 mm) was sequentially placed on a slide for Nissl staining. The area of stroke on each slice was viewed by light microscope (Nikon 80i, JPN) calculated using Image Pro-plus, and the volume was calculated by the following Eq. (1)^45^:

1 $${\text{Stroke}}\;{\text{volume}}\,(\mu m^{3} ) = \sum\limits_{{(1 - {\text{number}}\,{\text{of}}\,{\text{slices}})}} {[({\text{area}}\,{\text{of}}\,{\text{the}}\,{\text{contralesional}}\,{\text{hemisphere } - \text{ undamaged}}\,{\text{area}}\,{\text{of }}\,{\text{the }}\,{\text{ipsilesional}}\,{\text{hemisphere}})*30*10] }$$

### Immunofluorescence analysis

BrdU (50 mg/kg; B5002, Sigma-Aldrich, St. Louis, MO, USA) was injected intraperitoneally twice daily after surgery^34^, as indicated in Figs. 1a and 6a. The brain section at 20 μm were obtained as previous description. Every 10th section between bregma levels -0.30 and + 1.60 mm was selected (total = 3 sections per brain). For BrdU staining, the sections were permeabilized with 0.1 M phosphate-buffered saline (PBS) containing 0.3% Triton X-100 and incubated in 2 N HCl at 37 °C for 30 min before blocking in 3% donkey serum (017-000-121, Jackson ImmunoResearch Laboratories, Philadelphia, USA). Samples were then incubated overnight at 4 °C with the following primary antibodies: mouse anti-BrdU (1:200, 11170376001, Roche, Indianapolis, IN, USA), rabbit anti-Doublecortin (DCX) (1:200, ab18723, Abcam, Cambridge, MA, USA), rabbit anti-Nestin (1:200, ab92391, Abcam, Cambridge, MA, USA), or rabbit anti-NeuN (1:200, ab177487, Abcam, Cambridge, MA, USA). Appropriate Alexa Fluor 488- and Alexa Fluor 594-conjugated secondary antibodies were used to detect the primary antibodies (Alexa Fluor 488: A21206, Alexa Fluor 594: A21203, Life Technologies, Carlsbad, CA, USA). A mounting medium with 4,6-diamidino-2-phenylinidole (DAPI) (Abcam) was used. The fluorescence signals were visualized using a fluorescence microscope (Nikon 80i, JPN) or confocal microscope (TCS SP8, Leica, GER). All image analyses were conducted by observers who were blinded to the treatment conditions.

### Statistical analysis

Data were analyzed using SPSS 20.0 and expressed as the mean ± the standard error of the mean (SEM). For daily numbers of pasta retrieved in the pasta matrix reaching task poststroke, data were analyzed by repeated measures ANOVA. Distribution data from the pasta matrix reaching task were analyzed with the Mann–Whitney U-test. Binomially distributed data from each group were transformed to an approximately normal distribution using the following Eq. (2):

2 $$\begin{aligned} & {\text{Z}} = ({\text{P}}1 - {\text{P}}2)/({\text{P}}^{\prime } *(1 - {\text{P}}^{\prime } )*(1/{\text{n}}1 + 1/{\text{n}}2))^{1/2} \\ & {\text{P}}1 = {\text{X}}1/{\text{n}}1,\quad {\text{P}}2 = {\text{X}}2/{\text{n}}2,\quad {\text{P}}^{\prime } = ({\text{X}}1 + {\text{X}}2)/({\text{n}}1 + {\text{n}}2) \\ \end{aligned}$$

(X1: number of successful trials in population 1; X2: number of successful trials in population 2; n1: sample size for group 1; n2: sample size for group 2)^7^. The data for BrdU^+^/Nestin^+^ and BrdU^+^/DCX^+^ cells were compared between groups at 14 days and 21 days by Student's t-test. Other data were compared between groups by one-way ANOVA and Scheffe’s multiple comparison test. Please refer to figure legends for details on the statistics. Differences were considered significant at *P* < 0.05.

## Supplementary information

Supplementary Information.

## References

[1] Feigin VL (2018). Global, regional, and country-specific lifetime risks of Stroke, 1990 and 2016. N. Engl. J. Med..

[2] Lawrence ES (2001). Estimates of the prevalence of acute stroke impairments and disability in a multiethnic population. Stroke.

[3] Coscia M (2019). Neurotechnology-aided interventions for upper limb motor rehabilitation in severe chronic stroke. Brain.

[4] Elias GJB, Namasivayam AA, Lozano AM (2018). Deep brain stimulation for stroke: current uses and future directions. Brain Stimul..

[5] Hosomi K, Seymour B, Saitoh Y (2015). Modulating the pain network–neurostimulation for central poststroke pain. Nat. Rev. Neurol..

[6] Lindenberg R, Renga V, Zhu LL, Nair D, Schlaug G (2010). Bihemispheric brain stimulation facilitates motor recovery in chronic stroke patients. Neurology.

[7] Machado AG (2013). Chronic 30-Hz deep cerebellar stimulation coupled with training enhances post-ischemia motor recovery and peri-infarct synaptophysin expression in rodents. Neurosurgery.

[8] Shah AM (2017). Optogenetic neuronal stimulation of the lateral cerebellar nucleus promotes persistent functional recovery after stroke. Sci. Rep..

[9] Arvidsson A, Collin T, Kirik D, Kokaia Z, Lindvall O (2002). Neuronal replacement from endogenous precursors in the adult brain after stroke. Nat. Med..

[10] Thored P (2007). Long-term neuroblast migration along blood vessels in an area with transient angiogenesis and increased vascularization after stroke. Stroke.

[11] Palma-Tortosa S (2017). Specific features of SVZ neurogenesis after cortical ischemia: a longitudinal study. Sci. Rep..

[12] Sun FL (2014). Promoting neurogenesis via Wnt/beta-catenin signaling pathway accounts for the neurorestorative effects of morroniside against cerebral ischemia injury. Eur. J. Pharmacol..

[13] Liu T (2016). Morroniside promotes angiogenesis and further improves microvascular circulation after focal cerebral ischemia/reperfusion. Brain Res. Bull..

[14] Plow EB, Carey JR, Nudo RJ, Pascual-Leone A (2009). Invasive cortical stimulation to promote recovery of function after stroke a critical appraisal. Stroke.

[15] Nouri S, Cramer SC (2011). Anatomy and physiology predict response to motor cortex stimulation after stroke. Neurology.

[16] Omrani M, Murnaghan CD, Pruszynski JA, Scott SH (2016). Distributed task-specific processing of somatosensory feedback for voluntary motor control. Elife.

[17] Dum RP, Li C, Strick PL (2002). Motor and nonmotor domains in the monkey dentate. Ann. N. Y. Acad. Sci..

[18] Chan HH (2018). Lateral cerebellar nucleus stimulation has selective effects on glutamatergic and gabaergic perilesional neurogenesis after cortical ischemia in the rodent model. Neurosurgery.

[19] Chan HH (2018). Lateral cerebellar nucleus stimulation promotes motor recovery and suppresses neuroinflammation in a fluid percussion injury rodent model. Brain Stimul..

[20] Wathen CA, Frizon LA, Maiti TK, Baker KB, Machado AG (2018). Deep brain stimulation of the cerebellum for poststroke motor rehabilitation: from laboratory to clinical trial. Neurosurg. Focus.

[21] Zhao C, Deng W, Gage FH (2008). Mechanisms and functional implications of adult neurogenesis. Cell.

[22] Barker RA, Gotz M, Parmar M (2018). New approaches for brain repair-from rescue to reprogramming. Nature.

[23] Saha B, Peron S, Murray K, Jaber M, Gaillard A (2013). Cortical lesion stimulates adult subventricular zone neural progenitor cell proliferation and migration to the site of injury. Stem Cell Res..

[24] Sun FL (2014). Morroniside improves microvascular functional integrity of the neurovascular unit after cerebral ischemia. PLoS ONE.

[25] Wang W (2010). Neuroprotective effect of morroniside on focal cerebral ischemia in rats. Brain Res. Bull..

[26] Lin R (2018). Stepwise impairment of neural stem cell proliferation and neurogenesis concomitant with disruption of blood-brain barrier in recurrent ischemic stroke. Neurobiol. Dis..

[27] Vargas-Saturno L, Ayala-Grosso C (2018). Adaptive neurogenesis in the cerebral cortex and contralateral subventricular zone induced by unilateral cortical devascularization: possible modulation by dopamine neurotransmission. Eur. J. Neurosci..

[28] Jin K (2001). Neurogenesis in dentate subgranular zone and rostral subventricular zone after focal cerebral ischemia in the rat. Proc. Natl. Acad. Sci. USA.

[29] Tonchev AB (2011). Brain ischemia, neurogenesis, and neurotrophic receptor expression in primates. Arch. Ital. Biol..

[30] Dillen Y, Kemps H, Gervois P, Wolfs E, Bronckaers A (2020). Adult neurogenesis in the subventricular zone and its regulation after ischemic stroke: implications for therapeutic approaches. Transl. Stroke Res..

[31] Kreuzberg M (2010). Increased subventricular zone-derived cortical neurogenesis after ischemic lesion. Exp. Neurol..

[32] Dayer AG, Ford AA, Cleaver KM, Yassaee M, Cameron HA (2003). Short-term and long-term survival of new neurons in the rat dentate gyrus. J. Comput. Neurol..

[33] Zhang R (2004). Stroke transiently increases subventricular zone cell division from asymmetric to symmetric and increases neuronal differentiation in the adult rat. J. Neurosci..

[34] Morimoto T (2011). Striatal stimulation nurtures endogenous neurogenesis and angiogenesis in chronic-phase ischemic stroke rats. Cell Transpl..

[35] Vedam-Mai V (2014). Increased precursor cell proliferation after deep brain stimulation for Parkinson's disease: a human study. PLoS ONE.

[36] Chen YC (2017). Effects of anterior thalamic nuclei deep brain stimulation on neurogenesis in epileptic and healthy rats. Brain Res..

[37] Pohodich AE (2018). Forniceal deep brain stimulation induces gene expression and splicing changes that promote neurogenesis and plasticity. Elife.

[38] Schmuckermair C (2013). Behavioral and neurobiological effects of deep brain stimulation in a mouse model of high anxiety- and depression-like behavior. Neuropsychopharmacology.

[39] Lipp H, Bonfanti L (2016). Adult neurogenesis in mammals: variations and confusions. Brain Behav. Evol..

[40] Cooperrider J (2014). Chronic deep cerebellar stimulation promotes long-term potentiation, microstructural plasticity, and reorganization of perilesional cortical representation in a rodent model. J. Neurosci..

[41] Paxinos G, Watson C (1998). The Rat Brain in Stereotaxic Coordinate.

[42] Ballermann M, Metz GA, McKenna JE, Klassen F, Whishaw IQ (2001). The pasta matrix reaching task: a simple test for measuring skilled reaching distance, direction, and dexterity in rats. J. Neurosci. Methods.

[43] Metz GA, Whishaw IQ (2009). The ladder rung walking task: a scoring system and its practical application. J Vis Exp.

[44] Weston RM, Jarrott B, Ishizuka Y, Callaway JK (2006). AM-36 modulates the neutrophil inflammatory response and reduces breakdown of the blood brain barrier after endothelin-1 induced focal brain ischaemia. Br. J. Pharmacol..

[45] Roome RB (2014). A reproducible Endothelin-1 model of forelimb motor cortex stroke in the mouse. J. Neurosci. Methods.

