# Supplementary material for: Electrical stimulation of the lateral cerebellar nucleus promotes neurogenesis in rats after motor cortical ischemia
Source: Sci Rep. 2020 Oct 6;10:16563. doi: 10.1038/s41598-020-73332-5 (PMC7538419; doi:10.1038/s41598-020-73332-5)
Supplement: Supplementary file 1 — Supplementary Information. [file 41598_2020_73332_MOESM1_ESM.pdf]

Supplementary Information

Electrical Stimulation of the Lateral Cerebellar Nucleus promotes Neurogenesis in Rats after Motor Cortical Ischemia

Zheng Wu [1#], Fangling Sun [1#], Zijie Li [1], Min Liu [1], Xin Tian [1], Deyu Guo [3], Penghu Wei [2], Yongzhi Shan [2], Tingting Liu [1], Min Guo [1], Zixin Zhu [3], Wenrong Zheng [3], Yufeng Wang [3], Guoguang Zhao [2*], Wen Wang [1*]

1Department of Experimental Animal Laboratory, Xuan-wu Hospital of Capital Medical University, 45 Changchun Street, Beijing 100053, China;

2Department of Neurosurgery, Xuan-wu Hospital of Capital Medical University, 45 Changchun Street, Beijing 100053, China

3Department of Experimental Animal Laboratory, Beijing Geriatric Medical Research Center, 45 Changchun Street, Beijing 100053, China;

# Co-first authors

Corresponding authors: Guoguang Zhao (ggzhao@vip.sina.com) and Wen Wang (lzwwang@163.com)

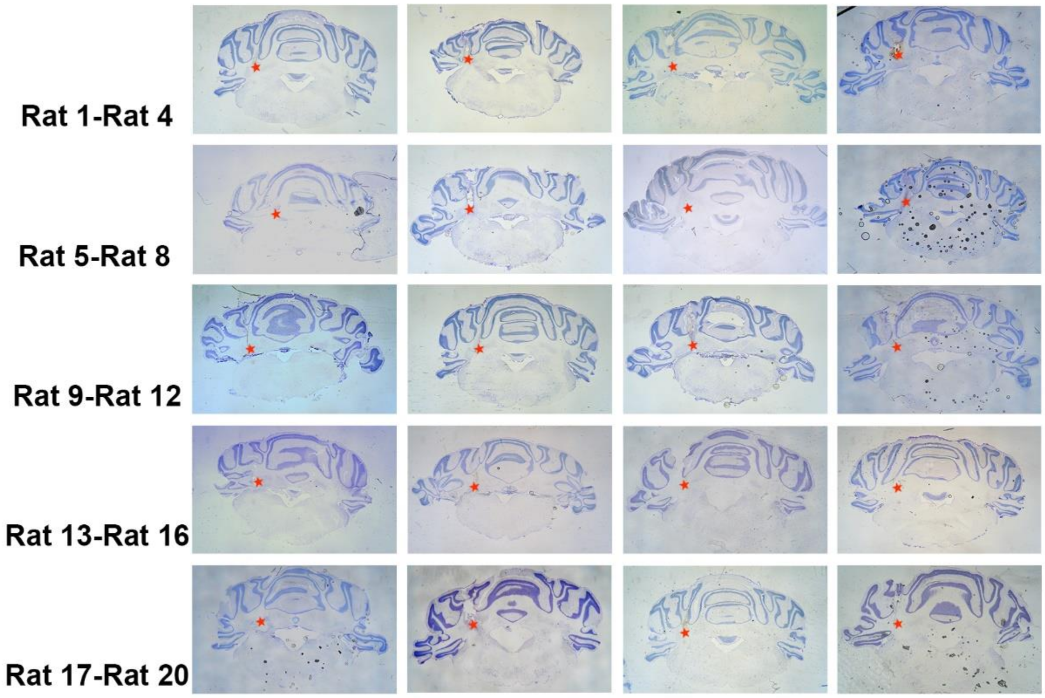

Supplementary Figure 1: Validation of LCN implant location by Nissl staining. Representative images of LCN implant location in each animal of ET-1+STIM group. ★ indicates the location of the implanted electrodes in the dorsal LCN.
